# Supplementary figures and images for: Safety and resource utilisation efficiency of semi-skeletonised versus skeletonised left internal mammary artery harvesting techniques: The BANGABANDHU study
Source: PLoS One. 2025 Sep 5;20(9):e0331285. doi: 10.1371/journal.pone.0331285 (PMC12412939; doi:10.1371/journal.pone.0331285)

**S1 Fig.** The goodness-of-fit of the logistic regression model.


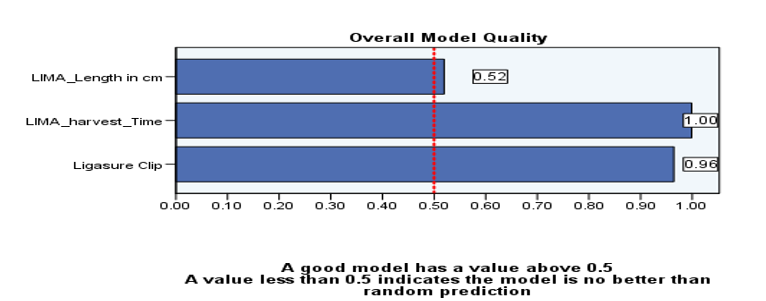

Supplement: S1 Fig — (DOCX) [file pone.0331285.s001.docx]
