# Supplementary material for: Safety and resource utilisation efficiency of semi-skeletonised versus skeletonised left internal mammary artery harvesting techniques: The BANGABANDHU study
Source: PLoS One. 2025 Sep 5;20(9):e0331285. doi: 10.1371/journal.pone.0331285 (PMC12412939; doi:10.1371/journal.pone.0331285)
Supplement: Attachment — Submitted filename: Response to Reviewers.docx [file pone.0331285.s002.docx]

**Dear Editor-in-Chief**

**PLOS ONE**

Thank you for considering our manuscript for publication in PLOS ONE. I am attaching our response to the reviewer comments and point-by-point clarifications for your review.

**Response to reviewer's comments**

**Reviewer #1:** I want to thank the authors for their research efforts and for the opportunity to review their manuscript. I have included some comments below followed by some line items.

**Q:** First, the three techniques would be skeletonised, semi-skeletonised and pedicled. Based on your description of semi-skeletonised, you need to explain how that is different than a pedicled LIMA (a pedicle also includes the veins, adipose, endothoracic fascia and muscle). I cannot tell the difference between semi-skeletonised and pedicled other than some arbitrary distance from the LIMA that is included in the dissection.

**Response:** I appreciate your concerns, which are not untrue. However, as a cardiac surgeon, you must recognise that minimising tissue damage during LIMA harvesting is crucial, especially since pedicled LIMA grafts are prone to sternal wound infections, particularly in patients with comorbidities. Therefore, we hypothesised that harvesting in a semi-skeletonised fashion, as you said, "some arbitrary distance from the LIMA that is included in the dissection", could be safe. Despite the lack of a standard definition, we defined the semiskelitonised technique in our study on page 7, lines 181-183 (yellow highlighted), which might be helpful in future studies to define semi-skeletonised LIMA.

**Q:** Second, you mention the length of the LIMA as a difference but you say that the protocol requires the same distance from proximal to the first intercostal branch to the bifurcation, so how is there a difference in length? In the results, the lengths need to be included. The difference was found to be significant at p=0.05 with the difference being 0.2 (which is inside the standard deviation so I am not sure how that is statistically significant). This seems to not be a important difference.

**Response:** Yes, we agreed, and in this study, we set a p-value of <0.05 as statistically significant, which we mentioned on page 8, lines 216,217 (yellow highlighted).

**Q:** Third, there is more than a doubling of time between skeletonised and semi-skeletonised. That seems to be excessive. This should be explored or explained a little more.

**Response:** While we mentioned it on page 10, lines 257-259 (highlighted in yellow), we have also added a brief explanation in lines 260-262 (highlighted in yellow).

**Q:** Fourth, you should make clear how many surgeons were included in this study. I believe you are saying that they all trained under the same surgeon. Is the current population from a single surgeon practice? Is it the CABG surgeon taking down the IMA or is it an assistant surgeon? This area of how many surgeons are participating is important to document.

**Response:** All study patients are from a single surgeon's practice, which we mentioned on page 6, lines 152,153. Further, the LIMA was harvested by two equally trained cardiac surgeons, which we mentioned in methodology sections lines 204-207.

**Q:** Line 36: "that evaluated 2209 age- and sex-matched adult Bangladeshi isolated CABG patients"

**Response:** Modifications are done as you suggested

**Q:** Line 38: I am not sure "curtailed" is the correct word here

**Response:** Thanks for your suggestions, "curtailed" replaced with "observed".

**Q:** Line 39: should be consistent and use "-"in semi-skeletonised or not (this is the first time it was not used)

**Response:** Modifications are done as you suggested

**Q:** Line 42: "we evaluated 2209 age- and sex-matched adult isolated CABG patients"

**Response:** Modifications are done as you suggested

**Q:** Line 44: should not capitalize anything other than EuroSCORE II and COPD

**Response:** Modifications are done as you suggested

**Q:** Line 45: usually, "p" is not capitalized (p> 0.05)

**Response:** Modifications are done as you suggested throughout the manuscript.

**Q:** Line 48: Furthermore, AN age- and sex-adjusted multivariate….

**Response:** Modifications are done as you suggested

**Q:** Line 52: you mention cost-effectiveness here without any discussion of it in the Results (you should not include a result in the Conclusion that was not presented in the Results)

**Response:** Conclusion modified as you suggested

**Q:** Line 86: "its blood supply" – do you mean the chest wall's blood supply? It is not clear in the way this sentence is written (the blood supply presumably refers to the chest wall and minimal surrounding tissue presumably refers to the LIMA but these two items are separated by an "and" which presumes both items are related to the same thing).

**Response:** The statement was modified as you suggested, with lines 84-86 (yellow highlighted).

**Q:** Line 95: "which is expensive" – I would say costs more money, but "expensive" is relative and in comparison with the cost of the entire operation, the Ligaclip is not a major driver of cost.

**Response:** Statement modified as you suggested, line 95 (yellow highlighted).

**Q:** Line 151: "We recruited 2209 age- and sex-matched adult CABG patients"

**Response:** Statement modified as you suggested, yellow highlighted

**Q:** Line 153: "We divided the study population into 2 groups based on…."

**Response:** Statement modified as you suggested, yellow highlighted

**Q:** Line 165: no need for the word "specifically"

**Response:** Statement modified as you suggested, yellow highlighted

**Q:** Line 179: when skeltonising, were Ligasure clips only used on the LIMA side just like in the semi-skeletonised scenario? This would be a difference in treatment if they were not the same.

**Response:** Yes. Ligaclips were usually used on the LIMA side in both the semi-skeletonised & skeletonised techniques which we mentioned in lines 179-181 (yellow highlighted).

**Q:** Line 186: "conduit is" – the paper has been written in the past tense, you switch to present here and then continue in the present tense in the next sentence.

**Response:** I appreciate your concern; line 193 (yellow highlighted) has been addressed and corrected.

**Q:** Line 193: "direct grasping of the LIMA with forceps was avoided by grasping nearby adventitial tissue."

**Response:** Modifications are done as you suggested, line 200 (yellow highlighted).

**Q:** Line 208: you switch from preterite to present here as well

**Response:** Modifications are done as you suggested, line 217, (yellow highlighted).

**Q:** Line 223: "evaluated 2209 age- and sex-matched adult Bangladeshi isolated CABG patients which included 1050 with a skeletonized LIMA and 1159 with a semi-skeletonised LIMA."

**Response:** Modifications are done as you suggested, lines 224-226 (yellow highlighted).

**Q:** Line 225: no words should be capitalized other than EuroSCORE II, COPD and CAD)

**Response:** Modifications are done as you suggested

**Q:** Line 227: you should include the lengths, times and number of clips here

**Response:** Modifications are done as you suggested, lines 228-230 (yellow highlighted).

**Q:** Line 228: "number of Ligaclips used"

**Response:** Modifications are done as you suggested

**Q:** Line 228: "compared to THE semi-skeletonised"

**Response:** Modifications are done as you suggested, line 231 (yellow highlighted).

**Q:** Line 231: AN age- and gender-adjusted

**Response:** Modifications are done as you suggested, line 233 (yellow highlighted).

**Q:** Line 232: "number of Ligaclips used"

**Response:** Modifications are done as you suggested

**Q:** Line 233: were significantly and positively associated with the semi-skeletonised LIMA technique

**Response:** Modifications are done as you suggested

**Q:** Line 234-236: I do not understand this sentence

**Response:** Sorry for the inconvenience. We have updated the statement as you suggested, and the changes are highlighted in yellow, lines 238-243.

**Q:** Line 236: this sentence needs to be re-worked'

**Response:** Statement modified as you suggested, yellow highlighted

**Q:** Line 284: this sentence needs to be re-worked

**Response:** Statement modified, lines 283-285 (yellow highlighted).

**Q:** Line 288: "is not"

**Response:** Modifications are done as you suggested, line 286 (yellow highlighted).

**Q:** Line 297: if the protocol was from proximal to the first intercostal branch to the bifurcation, then why was the skeletonised longer if both were prepared the same way in terms of length?

**Response:** We understand your concerns; however, the LIMA length was not significantly different between the study groups. This study was conducted because our senior consultants believe that a skeletonised LIMA provides more length, which was not the scenario in our study. However, the minimum length difference may be due to the adjacent fascia and veins associated with a semi-skeletonised LIMA rather than a skeletonised one.

**Q:** Line 302: I am not sure that this "mitigates the risk of outcome bias"

**Response:** We understand your concerns; however, every study has the potential for risk of bias, and we did our best to minimise the risk of bias by utilising two equally qualified surgeons to harvest the LIMA. Additionally, we changed "mitigates" to "minimises" in line 301 (highlighted in yellow).

**Reviewer #2:** This manuscript addresses a clinically relevant topic comparing semi-skeletonised and skeletonised LIMA harvesting techniques. However, several key issues limit its current suitability for publication.

**Q:** First, the authors emphasize cost-effectiveness but provide inadequate economic analysis. Simply counting Ligaclips is insufficient; a detailed cost breakdown including operative time, resource utilization, and hospital stay should be presented.

**Response:** We appreciate your concerns. The current study aimed to evaluate specific research questions regarding the safety and effectiveness of the LIMA harvesting technique rather than the whole CABG procedure, including hospital stay. Nevertheless, the overall operation cost was similar as all cases were performed by the same surgeon using off-pump techniques. However, we have revised our study objective statement for better clarity for our readers on page 2, and lines 31-33 (yellow highlighted).

**Q:** Second, the multivariate logistic regression model raises concerns. The odds ratios reported (e.g., OR 10.8 for LIMA harvest time) appear unusually large, suggesting potential errors in statistical modeling or interpretation. Clarification and re-analysis are needed.

**Response:** We agreed, and thank you very much for your suggestions. We revised the LR model by considering semi-skeletonised LIMA as a dependent variable. The modified OR values replaced the previous one on page 9, including an interpretation of our findings, lines 234-238 (yellow highlighted).

**Q:** Third, the authors mention early complications but do not adequately define or systematically evaluate them. Clear definitions, standardized follow-up periods, and explicit complication rates should be provided.

**Response:** This study evaluated specific research questions related to the intra-operative LIMA harvesting technique rather than the whole CABG procedure and follow-up outcomes. However, we modified the definition of study variables, including complications on page 6 and lines 168-172 (yellow highlighted).

**Q:** Lastly, the manuscript lacks clarity regarding surgeon experience standardization. Although a single surgeon's practice is mentioned, variability among trainees and junior surgeons may significantly confound results.

**Response:** Two equally qualified surgeons harvested LIMA who had completed postgraduate surgical training in cardiac surgery under a single-surgeon practice, which is mentioned on page 8 and lines 204-207 (yellow highlighted). However, we agreed that there might be a bias between surgeons, which we acknowledged as study limitations on page 11/12, lines 299-302 (yellow highlighted).

**Reviewer #3:** Dear Authors, Thank you for submitting your manuscript for review. I was pleased to receive it. Your study addresses a clinically relevant question and provides useful insights into the practical and cost-effective aspects of IMA harvesting techniques. However, I have some comments for your consideration:

**1.** In the abstract conclusion, you could briefly include clinical relevance or outcomes (e.g., complication rates, recovery, postoperative morbidity) alongside procedural efficiency.

**Response:** Thanks for your suggestion; however, an abstract conclusion should ideally be based on the most significant findings, which are short and precise. As you suggested, we modified our conclusive statement on page 2 (yellow highlighted).

**2.** You mention the "ambispective" nature of the study. Could you clarify how the prospective and retrospective data were integrated and managed to avoid biases regarding quality and completeness across the two collection approaches?

**Response:** We detailed the "ambispective" nature of the study on page 6, lines 158-167 (highlighted in yellow). Further, we have added a statement on how we integrated and managed quality and completeness across the two data collection approaches on page 6 and acknowledged a limitations statement on the risk of potential bias on page 12, lines 302-304 (yellow highlighted).

**3.** Why were certain covariates (e.g., hypertension, diabetes, COPD) matched but not directly included in the multivariate regression model? Could you explain this choice in the Methods?

**Response:** We detailed the selection of study variables for the regression model on page 8, lines 212-215 (highlighted in yellow).

**4.** Regarding the harvesting techniques, descriptions are clear. However, could you include additional intraoperative images (if available) to show differences between the techniques?

**Response:** Thanks, I appreciate your comments. We have added more pictures of skeletonised & semi-skeletonised LIMA, and Figure 1 has been modified as you suggested.

**5.** The use of Ligaclips as a cost indicator is well-justified. Still, could you provide an economic analysis or at least a brief comparative analysis of the overall economic impact (e.g., average procedural cost differences or estimated financial savings per case)?

**Response:** We appreciate your concerns. The current study aimed to evaluate specific research questions regarding the safety and effectiveness of the LIMA harvesting technique rather than the whole CABG procedure, including hospital stay. Nevertheless, the overall operation cost was similar as all cases were performed by the same surgeon using off-pump techniques. However, we have revised our study objective statement for better clarity for our readers on page 2, and lines 31-33 (yellow highlighted).

**6.** Your confidence intervals in the logistic regression (e.g., OR 10.8 with CI 0.01-0.39) seem incorrect in terms of directionality. Typically, OR >1 indicates increased odds. Please clarify.

**Response:** We agreed, and thank you very much for your suggestions. We revised the LR model by considering semi-skeletonised LIMA as a dependent variable. The modified OR values replaced the previous one on page 9, including an interpretation of our findings, lines 234-238 (yellow highlighted).

**7.** Can you compare semi-skeletonised and skeletonised techniques regarding long-term patency or graft survival, if this data is available?

**Response:** We apologise, but this study focuses specifically on evaluating the LIMA harvesting technique; unfortunately, we do not have long-term follow-up data on patency or graft survival. However, in response to your suggestions, we have included a recommendation for future studies to validate the robustness of our findings on page 12, lines 314-316 (yellow highlighted).

**8.** Given that all surgeries were performed by qualified surgeons within a single surgeon's practice, how generalizable do you believe these results are? Could you briefly discuss how the familiarity of surgeons with each technique may influence results?

**Response:** We understand your concerns; however, Professor Adhikary is one of the most senior CTh surgeons, with over 30 years of experience in CABG surgery. He has trained surgeons at the only medical university hospital in Bangladesh, which is why we believe the outcome results are generalisable despite the inherent risk of bias that is standard in any observational study. Additionally, in this study, the LIMA was harvested by two equally trained cardiac surgeons, as noted on page 8, lines 204-207 (highlighted in yellow). However, we agreed that there might be a bias between surgeons, which we acknowledged as study limitations on page 11/12, lines 299-302 (yellow highlighted).

**9.** You have mentioned the identical comorbidities between study groups, demonstrating good baseline comparability. However, given the single-center, single-surgeon practice setting, could there be any institutional protocols or perioperative management peculiarities that may have influenced outcomes differently from broader multi-institutional contexts? Thank you again for the opportunity to review your work.

**Response:** Bangladesh has no standard institutional guidelines for LIMA harvesting techniques. We believe that there are no specific factors related to perioperative management that uniquely affect the outcomes of different LIMA harvesting techniques. In this study, we addressed specific questions regarding the safety and benefits of the semi-skeletonised LIMA harvesting technique compared to the skeletonised LIMA technique.

Thank you very much for your understanding; we appreciate your comments.

We hope this version meets your standards for publication approval.

With thanks and regards

Dr Redoy Ranjan

**Associate Editor, PLOS ONE	Institute of Cardiovascular Research, Royal Holloway University of London, London, UK** Department of Cardiac Surgery, St George’s University Hospitals NHS Foundation Trust, UK	Email: [redoy_ranjan@](mailto:redoy_ranjan@yahoo.com)bsmmu.edu.bd
