# Supplementary material for: Safety and resource utilisation efficiency of semi-skeletonised versus skeletonised left internal mammary artery harvesting techniques: The BANGABANDHU study
Source: PLoS One. 2025 Sep 5;20(9):e0331285. doi: 10.1371/journal.pone.0331285 (PMC12412939; doi:10.1371/journal.pone.0331285)
Supplement: Attachment — Submitted filename: R2_Response Letter.docx [file pone.0331285.s003.docx]

**Dear Editor-in-Chief**

**PLOS ONE**

Thank you for considering our manuscript for publication in PLOS ONE. I am attaching our response to the reviewer comments and point-by-point clarifications for your review.

**Response to reviewer's comments**

**Reviewer #1:** I want to thank the authors for their thorough responses to prior comments. I have one comment below followed by some line items.

1. I am still concerned that the central item around which the entire paper revolves is the “semi-skeletonized” LIMA and you are unable to provide a definition that distinguishes it from the only other most commonly used harvest technique which is the pedicled technique. The two most common techniques are skeletonized and pedicled and so it is glaring that this paper does not mention the pedicled technique in any substantial or comparative way. In order to do that, you would have to distinguish the semi-skeletonized and the pedicled and I do not know how you would distinguish those. As you mention, as a cardiac surgeon, they all recognize that minimizing tissue damage during LIMA harvesting is crucial so pedicled LIMAs are usually small (surgeons shy away from taking a large swath of chest wall when taking the pedicle) so how small is small to qualify as semi-skeletonized as opposed to a small pedicle? I think you need to try an attempt at distinguishing the semi-skeletonized vs a pedicled LIMA since even though you do not bring up pedicled LIMAs (other than a few sentences in the Discussion), every surgeon reading this will be wondering what makes your technique different than theirs (if they take small pedicles).

**Response:** Thanks for your critical appraisal. We have briefly described the semi-skelitonised LIMA technique with appropriate in-text citations (lines 206-212; New Ref number 17). In brief, Horii and Suma have described the semiskeletonised LIMA as a venoarterial pedicle with surrounding thin tissue without muscular support, and keeping the endothoracic fascia attached to the chest wall.

**Line 57-60:** I am not sure what section this belongs to, but there needs to be a reference noted for all these claims

**Response:** We understand your concerns. However, as the PLOS One manuscript guidelines don't include those sections, we have deleted the "**What is already known on this topic? What this study adds? & How might this study affect research, practice or policy?"** sections.

**Line 102:** to what drainage are you referring?

**Response:** Apologies for the inconvenience. The sentence has been modified, as highlighted in yellow on line 124.

**Line 154-155:** this should be mentioned in limitations that you divided (did not randomize) the group into how the LIMA was harvested. It is possible that a significant sorting mechanism was already operating when the patient was chosen for skeletonized vs semi-skeletonized.

**Response:** Appreciate your comments. There was no sorting mechanism; the LIMA harvesting was conducted randomly. We have modified statements as you suggested, lines 176 & 177, highlighted in yellow.

**Line 180:** the word “should” should not be here – you are describing what happened, you are not explaining how to do it

**Response:** Thank you for your attention to detail. We modified the statement as you suggested in line 202 (highlighted in yellow).

**Line 280:** you are now comparing semi-skeletonized to pedicled – I am not sure of the relevance to this paper as you have not discussed pedicled LIMAs. What is the drainage compared to the skeletonized (the comparison in this paper)?

**Response:** We understand and appreciate your concerns, so we have removed line 280, as pedicle LIMA is irrelevant to this paper.

**Reviewer #2:** The authors' revisions significantly improve manuscript clarity, but critical issues persist.

**Firstly,** the semi-skeletonised technique remains inadequately defined, described ambiguously as "some arbitrary distance" from LIMA, raising reproducibility concerns. The authors' reply partially addresses this but needs explicit anatomical landmarks for precision.

**Response:** Thanks for your critical appraisal. We have added a brief description of the semi-skelitonised LIMA technique as previously described elsewhere with appropriate in-text citations (lines 206-212; New Ref number 17).

**Secondly,** statistical interpretation remains problematic. Despite revision, the statistical significance of minimal length differences (0.2 cm) within the standard deviation requires clearer clinical justification, potentially representing type I error.

**Response:** We defined P <0.05 as significant (not P =0.05), but acknowledge your concerns about P =0.05 being borderline significant, which can occur with a small mean ±SD difference due to the large sample size, and low variability in LIMA length as mean (SD) decreases with increasing sample size and low variability in data, making even minor differences more detectable. However, we acknowledged and discussed these issues in the limitations section with appropriate in-text citations (lines 332-335).

**Thirdly,** the doubled operative time in the skeletonised group requires deeper analysis beyond superficial acknowledgment; underlying procedural variations or surgeon-specific factors should be clarified further.

**Response:** We understand your concerns. However, there were no procedural variations as all cases were performed using off-pump CABG techniques and no surgeon-specific factors influenced LIMA harvesting time as all surgeons or trainees harvested both skeletonised and semi-skeletonised LIMA, which we mentioned on page 8, line 233-236.

**Lastly,** economic analysis remains superficial, relying solely on Ligaclip counts without comprehensive cost breakdown. Recommend: revise thoroughly to address definition precision, statistical robustness, detailed procedural explanations, and comprehensive economic analysis.

**Response:** Thanks for your suggestions; however, as I said earlier, comprehensive economic analysis was beyond the study objectives, as the current study aimed to compare the 2 LIMA harvest techniques in OPCABG surgery. Further, we modified the “cost-effectiveness” term and replaced it with “resource utilisation efficiency” throughout the manuscript as per the suggestions of Reviewer 3 (yellow highlighted). Nonetheless, we have added a recommendation to conduct further research on the cost-effectiveness of two harvesting techniques, considering factors such as total procedural costs, length of hospital stay, and follow-up expenses (lines 326-329; highlighted in yellow).

**Reviewer #3:** Dear Authors, Thank you for your thorough responses and revisions. The manuscript has been significantly improved, and many prior concerns have been addressed. I have only two further suggestions:

1. The term “cost-effectiveness” may overstate the economic implications of your study given the absence of broader economic analyses (e.g., total procedural costs, hospital stay, follow-up costs). Consider revising this term to “procedural efficiency” or “resource utilization efficiency” throughout the manuscript, including the title and abstract.

**Response:** Thanks for your suggestions; we modified the “cost-effectiveness” term and replaced it with “resource utilisation efficiency” throughout the manuscript as per your suggestions (yellow highlighted). Nonetheless, we recommend conducting a further study to evaluate the cost-effectiveness of two harvesting techniques, taking into account the details of total procedural costs, length of hospital stay, and follow-up expenses, etc., as you suggested (lines 326-329; yellow highlighted).

2. The clinical relevance of early complications (e.g., LIMA spasm, hematoma, re-exploration) is not sufficiently discussed. For instance, you could note the low incidence of these events and the absence of significant between-group differences to support the safety profile of the semi-skeletonized technique. Thank you again for your efforts.

**Response:** Thanks for your suggestions, we have added a brief discussion on these issues on page 11, lines 306-309 (yellow highlighted).

We hope this revised version meets your standards for publication approval.

With thanks and regards

Dr Redoy Ranjan

**Associate Editor, PLOS ONE	Institute of Cardiovascular Research, Royal Holloway University of London, London, UK** Department of Cardiac Surgery, St George’s University Hospitals NHS Foundation Trust, UK	Email: [redoy_ranjan@](mailto:redoy_ranjan@yahoo.com)bsmmu.edu.bd
