# Supplementary material for: Safety and resource utilisation efficiency of semi-skeletonised versus skeletonised left internal mammary artery harvesting techniques: The BANGABANDHU study
Source: PLoS One. 2025 Sep 5;20(9):e0331285. doi: 10.1371/journal.pone.0331285 (PMC12412939; doi:10.1371/journal.pone.0331285)
Supplement: Attachment — Submitted filename: R3_Response letter.docx [file pone.0331285.s004.docx]

**Dear Editor-in-Chief**

**PLOS ONE**

Thank you for considering our manuscript for publication in PLOS ONE. I am attaching our response to the reviewer comments and point-by-point clarifications for your review.

**Re:** Response to Review Comments on PONE-D-25-07678R2

**Reviewer #1:** I want to thank the authors for their responses. Overall, I have a couple of items left.

**1.** The last sentence of the discussion says “cost-effectiveness” and this word should be changed as this study is not a cost-effectiveness analysis.
**Response:** Thank you very much for your attention to detail. The statement has been modified as you suggested, with the changes highlighted in yellow (line 337).

**2.** Unless skeletonized vs semi-skeletonized were chosen by a randomizing mechanism, the claim that the use of either technique was randomized is not accurate and should be removed. Without a randomizing mechanism, the choice is up to the surgeon and they choose which intervention to give and that is not accepted as being randomized.

**Response:** We appreciate your constructive comments. Although we randomly performed either skeletonised or semi-skeletonised techniques, we agreed that the sampling procedures didn't comply with the full structure of a randomised trial, which is why we modified the sentences as you suggested (highlighted in yellow, line 177).

**Reviewer #2:** Despite these improvements in the revision, several critical issues remain unresolved:
**1.** The “semi-skeletonised” LIMA technique is still vaguely defined. The revision cites Horii’s description of a thin venoarterial pedicle but omits precise anatomical boundaries. Without clear landmarks, the method is clearly indistinguishable from a minimal pedicled harvest, undermining reproducibility.
**Response:** Dear Reviewer, to the best of our knowledge, Horii's description of semi-skeletonised LIMA is precise and clear, and we have precisely defined the anatomical boundaries in lines 204-212 & 215-218 (yellow highlighted). Unfortunately, neither do we have an idea nor have we found literature defining "minimal pedicled harvest" techniques.

**2.** A 0.2 cm LIMA length difference (p≈0.05) is statistically marginal and clinically negligible. Treating this borderline result as significant overstates its importance and likely reflects sample-size effects.

**Response:** We agree that a p-value of 0.05 is not statistically significant, as noted in the methodology section. A p-value of <0.05 was considered statistically significant (highlighted in the yellow, lines 244 & 245). However, we utilise independent variables with a p-value of ≤0.05 in the multivariate LR model to avoid missing potentially important effects, e.g. false negative. Nevertheless, we have already acknowledged your concerns as a study limitation in lines 332-335, highlighted in yellow with appropriate citations.

**3.** The skeletonised harvest time was double the semi-skeletonised time, yet this is unexplained. Claiming uniform technique and staff ignores potential learning-curve or procedural complexity factors behind such a large efficiency gap.

**Response:**  We understand your concerns, which we have already explained in the discussion section (Yellow highlighted, lines 282-285). Furthermore, the difference in harvesting time may be associated with the learning curve or procedural complexity; however, we confirmed that all surgeons were equally qualified and performed both harvesting techniques (Yellow highlighted, lines 233-236). Despite the risk of bias, a large sample like ours minimises the risk of bias and confirms the generalisability of the findings.

**4.** No substantive economic analysis is presented. Merely counting Ligaclips and renaming outcomes as “resource utilization” falls short of a genuine cost comparison, contrary to the manuscript’s framing.

**Response:** We appreciate your concerns; however, in LMICs like Bangladesh, saving ~25 ligaclip per case indicates significant savings at the end of the year. For example, 25 Ligaclips per case saves approximately $100, which means that, annually in Bangladesh, we perform around 8,000 CABG surgeries, resulting in a potential annual savings of approximately $800,000.00. This is a significant amount for both the Bangladeshi people and the government. I understand your work in the NHS, which is well-organised and fully supported by the UK government; however, a simple modification of the skeletonised technique might help people in LMICs significantly.

However, as you raised concerns and as 3rd reviewer suggested, "cost-effectiveness" terms were replaced with "resource utilisation", and in the absence of a comprehensive economic analysis, we recommended further research on the cost-effectiveness of two harvesting techniques, considering factors such as total procedural costs, length of hospital stay, and follow-up expenses (lines 326-329; highlighted in yellow).

**Reviewer #3:** Dear Authors,

Thank you for considering my suggested revisions. Overall, you have put a lot of effort into revising your work by integrating the feedback provided. I believe the resulting changes have significantly improved the rigor and overall quality of your manuscript.
Congratulations on your work.

**Response:** Thank you very much for your constructive comments and valuable suggestions, which greatly enhance the manuscript's quality.

We hope this revised version meets your standards for publication approval.

With thanks and regards

Dr Redoy Ranjan

**Associate Editor, PLOS ONE	Institute of Cardiovascular Research, Royal Holloway University of London, London, UK** Department of Cardiac Surgery, St George’s University Hospitals NHS Foundation Trust, UK Department of Cardiac Surgery, Bangabandhu Sheikh Mujib Medical University, Bangladesh	Email: [redoy_ranjan@](mailto:redoy_ranjan@yahoo.com)bsmmu.edu.bd
