# Supplementary material for: Safety and resource utilisation efficiency of semi-skeletonised versus skeletonised left internal mammary artery harvesting techniques: The BANGABANDHU study
Source: PLoS One. 2025 Sep 5;20(9):e0331285. doi: 10.1371/journal.pone.0331285 (PMC12412939; doi:10.1371/journal.pone.0331285)
Supplement: Attachment — Submitted filename: Response letter_PONE-D-25-07678R3.docx [file pone.0331285.s005.docx]

Dear Editor-in-Chief

PLOS ONE

Thank you for considering our manuscript for publication in PLOS ONE. I am attaching our response to the reviewer comments and point-by-point clarifications for your review.

**Re:** Response to Review Comments on PONE-D-25-07678R3

**Reviewer #1:** I want to thank the authors for answering my questions. I believe that the paper is much better and helps correctly place the findings as the outcomes of a single institutional experience. I have a couple general comments below followed by some line items.

**1.** First, it should be acknowledged that your institutional preference is use Ligaclips, but many other surgeons use clips on reusable clip appliers and when using this latter method, the cost difference is likely negligible.

**Response:** We appreciate your concerns; however, in Bangladesh, all surgeons and institutes utilise Ligaclips with reusable clip applicators, which I mentioned in line 252. Further, I believe you overlooked my explanation in the previous version, as the same concerns were raised by Reviewer 2.

Although "the cost difference is likely negligible", per case, like you said, saving ~25 ligaclip per case indicates significant savings at the end of the year. For example, 25 Ligaclips per case saves approximately $100, which means that, annually in Bangladesh, we perform around 8,000 CABG surgeries, resulting in a potential annual savings of roughly $800,000.00. This is a significant amount for both the Bangladeshi people and the government. I appreciate your work at a well-organised institute supported by the government; however, a simple modification of the skeletonised LIMA technique might significantly help people in LMICs.

**2.** Second, were these all primary operations or were some redo’s? This would provide a major source of bias and so it should be addressed.

**Response:** This study included isolated primary (elective and emergency) CABG procedures, including redo CABG cases if the LIMA is available for harvesting. I have revised the methodology section as you recommended for better clarity on pages 6 and 8, specifically lines 175-176 and lines 249-251. For both primary and redo CABG, the harvesting techniques for the LIMA remain consistent, as outlined in the LIMA harvesting technique section on pages 7-9. This consistency helps mitigate the risk of bias. Additionally, we have added a limitations statement regarding this concern on page 13, lines 361-364.

**3.** Line 205-209: it is a little unclear here with the first sentence stating “semi-skeletonised LIMA technique preserved……” followed by the subsequent sentence stating “semi-skeletonized LIMA was excised as…..” which included structures that it said were preserved in the prior sentence. These two sentences can be written more clearly to let the reader know what the exact technique was.
**Response:** We apologise for the inconvenience; we have revised the statement for improved clarity and presentation in lines 219-222.

**4.** Line 219: “we tried to avoid opening the pleural space”. Line 231: “in both cases, we opened the left pleura to enhance LIMA visualization”. These are contradictory statements.

**Response:** Thank you for your careful attention to detail. To improve clarity, we have removed line 219, which stated, "we tried to avoid opening the pleural space." We have retained line 231 (now line 243): "In both cases, we opened the left pleura to enhance LIMA visualisation."

**5.** Line 237: you have to remove the word “randomly” here – it was not randomized. You changed it in Line 177 and it needs to be changed here too.
**Response:** We have now replaced "randomly" with "stochastically" on line 249; the change is highlighted in yellow.

**6.** Line 286: “However” seems to refer to the prior sentence (semi-skeletonized) and the “However” sentence seems to refer to the skeletonized technique so the two sentences are combined together with “however” incorrectly.
**Response:** Thank you for your attention to detail. The statement has been modified for improved clarity and presentation, as indicated in line 310 (highlighted in yellow).

**7.** Line 308: What was your definition of hematoma? Leaking of vasovasorum over a certain distance? Impeding blood flow? And what was your definition of spasm?
**Response:** There is currently no standard distance threshold that defines a LIMA hematoma or spasm. I have briefly described the LIMA hematoma and spasm in lines 196-209. Additionally, I included the current study strategies for managing cases of LIMA hematoma or spasm and outlined our approach to dealing with these issues.

**8.** Line 330: I would leave out “favoring semi-skeletonized techniques” – you recommend doing future studies and you do not know the outcomes (hence the need for the studies) so you should not say that they are going to favor one technique or the other.

**Response:** I appreciate your attention to detail. As you suggested, I have removed "favouring semi-skeletonised techniques" from lines 355.

**Reviewer #2:** The authors have diligently addressed the previous reviewers' concerns.
The manuscript has been revised to replace "cost-effectiveness" with the more accurate "resource utilisation efficiency," and the inaccurate claim of randomization was corrected to "stochastically performed." The authors clarified the surgical technique by providing more precise anatomical descriptions and procedural details. They also appropriately acknowledged the marginal statistical significance of the LIMA length difference as a study limitation. The statistical analysis is sound, and the English is suitable for publication. The authors provided compelling arguments for their findings, particularly regarding the significant differences in harvesting time and clip usage between the two methods.

**Response:** We sincerely thank the reviewer for their kind words and thoughtful evaluation. We appreciate the recognition of the attention to detail in our work, and we are grateful for the positive feedback and encouragement. It reinforces our commitment to maintaining high standards in both methodology and presentation.

**Reviewer #3:** Dear Authors,

Although I did not provide specific suggestions in the previous round, I appreciate the revisions you made in response to the comments from the other reviewers. These changes have further strengthened the manuscript and clearly positioned it as a resource utilization analysis.
I wish you every success with the publication of your work.

**Response:** We sincerely thank the reviewer for their thoughtful and encouraging comments. We greatly appreciate your recognition of the strengths of our work and your emphasis on the importance of attention to detail. Your positive feedback reinforces our commitment to scientific rigour, and we have carefully reviewed the manuscript to ensure consistency, clarity, and precision throughout.

We hope this revised version meets your standards for publication approval.

With thanks and regards

Dr Redoy Ranjan

**Associate Editor, PLOS ONE	Institute of Cardiovascular Research, Royal Holloway University of London, London, UK** Department of Cardiac Surgery, St George’s University Hospitals NHS Foundation Trust, UK Department of Cardiac Surgery, Bangabandhu Sheikh Mujib Medical University, Bangladesh	Email: [redoy_ranjan@](mailto:redoy_ranjan@yahoo.com)bsmmu.edu.bd
